# Assessment of Iodine Contrast-To-Noise Ratio in Virtual Monoenergetic Images Reconstructed from Dual-Source Energy-Integrating CT and Photon-Counting CT Data

**DOI:** 10.3390/diagnostics12061467

**Published:** 2022-06-14

**Authors:** Ronald Booij, Niels R. van der Werf, Marcel L. Dijkshoorn, Aad van der Lugt, Marcel van Straten

**Affiliations:** Department of Radiology & Nuclear Medicine, Erasmus University Medical Center, 3015 GD Rotterdam, The Netherlands; nrvdwerf@gmail.com (N.R.v.d.W.); m.l.dijkshoorn@erasmusmc.nl (M.L.D.); a.vanderlugt@erasmusmc.nl (A.v.d.L.); marcel.vanstraten@erasmusmc.nl (M.v.S.)

**Keywords:** X-ray computed tomography, iodine, dual-energy, photon-counting CT, image quality

## Abstract

To evaluate whether the contrast-to-noise ratio (CNR) of an iodinated contrast agent in virtual monoenergetic images (VMI) from the first clinical photon-counting detector (PCD) CT scanner is superior to VMI CNR from a dual-source dual-energy CT scanner with energy-integrating detectors (EID), two anthropomorphic phantoms in three different sizes (thorax and abdomen, QRM GmbH), in combination with a custom-built insert containing cavities filled with water, and water with 15 mg iodine/mL, were scanned on an EID-based scanner (Siemens SOMATOM Force) and on a PCD-based scanner (Siemens, NAEOTOM Alpha). VMI (range 40–100 keV) were reconstructed without an iterative reconstruction (IR) technique and with an IR strength of 60% for the EID technique (ADMIRE) and closest matching IR strengths of 50% and 75% for the PCD technique (QIR). CNR was defined as the difference in mean CT numbers of water, and water with iodine, divided by the root mean square value of the measured noise in water, and water with iodine. A two-sample t-test was performed to evaluate differences in CNR between images. A *p*-value < 0.05 was considered statistically significant. For VMI without IR and below 60 keV, the CNR of the PCD-based images at 120 and 90 kVp was up to 55% and 75% higher than the CNR of the EID-based images, respectively (*p* < 0.05). For VMI above 60 keV, CNRs of PCD-based images at both 120 and 90 kVp were up to 20% lower than the CNRs of EID-based images. Similar or improved performance of PCD-based images in comparison with EID-based images were observed for VMIs reconstructed with IR techniques. In conclusion, with PCD-CT, iodine CNR on low energy VMI (<60 keV) is better than with EID-CT.

## 1. Introduction

The number of computed tomography (CT) scans annually performed is still increasing [1,2]. Most of them are performed with iodinated contrast agents [3]. A low tube voltage is often used in contrast-enhanced CT to increase low-contrast detectability, or to allow lower radiation exposure with a similar contrast-to-noise ratio (CNR) [4]. CT scanners capable of acquiring dual-energy data with different approaches, e.g., dual-source CT (DSCT), rapid tube voltage switching X-ray sources and dual-layer detector technology, can offer virtual monoenergetic images (VMI) at arbitrary energy levels (in keV), derived from energy resolved attenuation data [5,6,7]. Iodine versus soft tissue contrast is enhanced at reduced energy levels, especially at energies of 40 to 70 keV, thanks to the relatively large increase in X-ray attenuation by iodine when lowering the X-ray energy. 

Unlike energy-integrating detectors (EID), a photon-counting detector (PCD) allows for photon energy discriminating measurements by counting the number of photons within predefined energy bins [8,9]. Thus, spectral data are acquired during every scan, which enables the reconstruction of VMI CT images by default. The comparison of CNR-levels in VMI images at equal monoenergetic levels is mostly a comparison of noise levels. The noise in VMI images, especially at low keV, is greatly affected by the noise reduction algorithm that utilizes the redundancy in low and high energy measurements. Acquiring energy-resolved attenuation measurements with a PCD-based system operating at a single tube voltage will affect the spectral separation. On the other hand, compared to DSCT with an energy-integrating detector, it offers the ability to use energy weighting and it registers photons of different energies at exactly the same position in time and space. 

Studies with prototypes of PCD-based CT scanners of several manufacturers illustrated, besides increased spatial resolution and structural visualization, CNR improvements over conventional CT systems using EID [10,11,12,13,14]. Sawall et al. showed that, prior to the application of vendor-specific noise reduction algorithms, the CNR in PCD-based iodine images is similar to the CNR in EID-based images [15]. Recently, PCD-CT has been introduced in the clinic for whole-body imaging [16]. Euler et al. proposed VMI at 50 keV with this system to obtain an increased CNR compared with EID-CT [17]. Higashigaito et al. indicate that VMI at 50 keV also shows significantly higher CNR [18]. The latter two studies, however, do not compare VMI in PCD-CT with VMI in EID-CT, but with conventional polychromatic EID-CT. The clinical PCD-CT system comes with a novel iterative reconstruction algorithm that also improves CNR [19,20]. The net effect on the CNR of PCD-based VMI, including vendor-specific noise reduction and iterative reconstruction algorithms, in comparison with state-of-the-art VMI with an EID-CT system, remains unknown and is the subject of this study. 

The purpose of this study is to evaluate whether the CNR of iodinated contrast agents in VMI from the first clinical PCD-based CT scanner differs from the CNR in VMI from a dual-source dual-energy CT scanner with conventional EID, while taking the influence of iterative reconstruction techniques into account.

## 2. Materials and Methods

### 2.1. Phantoms and CT Systems

**Figure 1:**
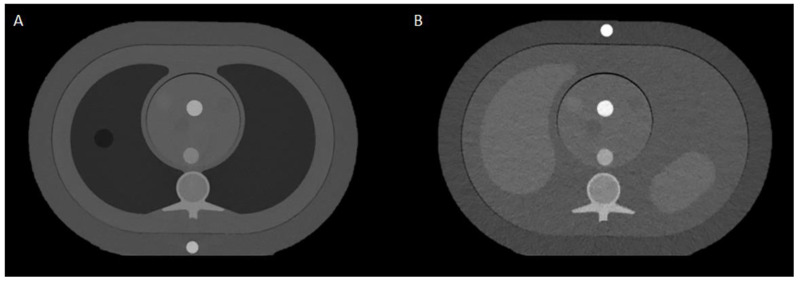
CT Images of the thoracic (**A**) and the abdominal (**B**) phantom with the medium size extension ring and the insert containing the iodine and water tubes.

Two anthropomorphic phantoms (QRM Thorax and QRM Abdomen, QRM GmbH) in combination with an insert containing water and 15 mg iodine/mL (Figure 1) were scanned on an EID-based dual-source CT (DSCT) system (SOMATOM Force, Siemens Healthineers, Syngo CT VB20, Forchheim, Germany) and the first clinical PCD-based CT system (NAEOTOM Alpha, Siemens Healthineers, Syngo CT version VA40A.2.01) [16]. Phantom dimensions were increased by two fat tissue equivalent extension rings (QRM-extension ring 350 × 250 mm and 400 × 300 mm, QRM GmbH) for both phantoms to resemble a medium (M) and large (L) patient size, respectively. When no extension ring was used, the patient size was considered small (S) [21].

### 2.2. Data Acquisition and Reconstruction Parameters

Acquisition and reconstruction parameters are listed in Table 1. Clinically-used dose reference values were applied to determine the typical CTDIvol values for all phantom types and sizes. For the EID-based system, the data were acquired while operating at two different tube voltages simultaneously in dual-source mode. Additional tin (Sn) filtration was applied to the X-ray tube operating at the highest peak voltage. For the PCD-based system, two scans were acquired with a single tube voltage (120 kVp and 90 kVp) and in single-source mode. All scans were repeated three times. For a fair comparison, the same reconstruction kernel (Qr40) with similar modulation transfer characteristics was used for both scanners and reconstructed with and without iterative (IR) reconstruction. Images for the EID-based system were reconstructed with the advanced modeled iterative reconstruction (ADMIRE, Siemens Healthineers) and weighted-filtered-back-projection (WFBP). Quantum iterative reconstruction (QIR, Siemens Healthineers) and “QIR-off” were used to reconstruct the images of the PCD scanner, as standard WFBP images on a PCD-based scanner are not applicable in the reconstruction process. As ADMIRE has 5 different strengths; the often used clinically and middle strength setting of IR was chosen (strength 3; 60%). As QIR has four different strengths, the two closest matching IR strengths, i.e., 2 (50%), and 3 (75%), were used. A QIR strength of 75% is often used in clinical routine. 

### 2.3. Data Analysis

For each scan, the CNR was determined by placing regions of interest (ROI) in six consecutive images of the tubes containing water and water with iodine. For each tube, mean and standard deviation of the CT numbers (expressed in Hounsfield units (HU)) in the ROIs were measured and averaged over all images. CNR was defined as the difference in mean CT numbers of water and water with iodine, divided by the root mean square value of the measured noise in water and water with iodine. The computations were performed with an in-house developed script (MATLAB R2015b, The MathWorks Inc., Natick, MA, USA). At each monoenergetic energy level, the CNR-value was compared between EID-based CT and PCD-based CT for all tube voltages and reconstruction techniques applied. 

### 2.4. Statistical Analysis

MATLAB (version R2015b, The MathWorks Inc.) was used for statistical analysis. CNR-values are given as mean values of the three measurements. The two-sample t-test was performed to evaluate statistically significant differences in the CNR-values between reconstructed scans. The test was conducted while assuming equal variances for the CNR-values. A *p*-value < 0.05 was considered statistically significant. Approximately 95% confidence limits for the percentage change in CNR-value were obtained via propagation of the standard errors (SE) of the mean CNR-values.

## 3. Results

### 3.1. CNR

**Figure 2:**
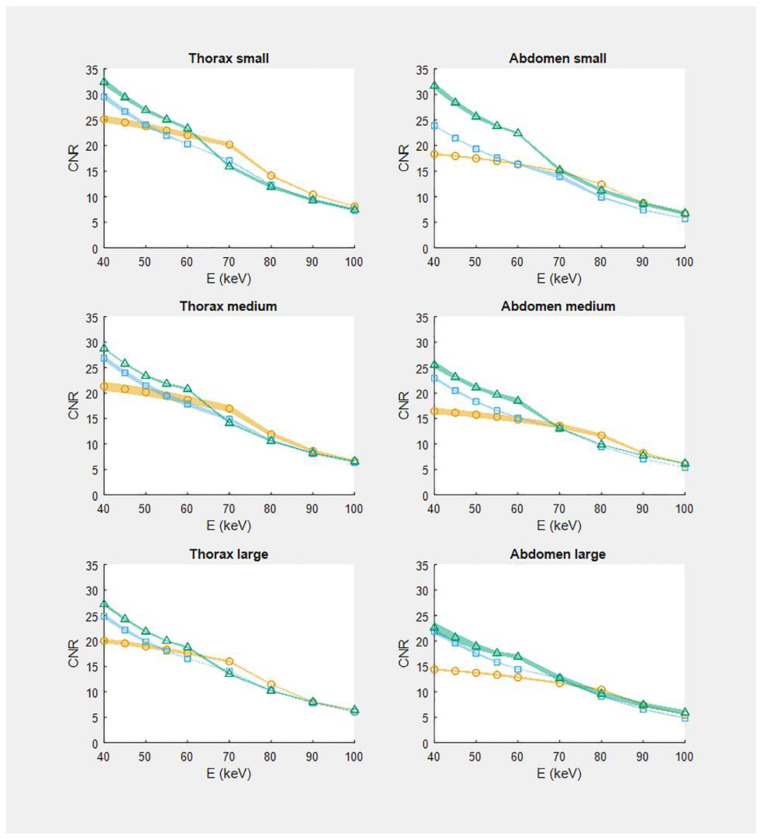
CNR in virtual monoenergetic images reconstructed without iterative reconstruction technique as a function of energy E (keV). For each phantom, CNR is presented for both the EID and PCD-based system. Circles: dual-source EID-based dual-energy; squares: single-energy 120 kVp PCD-CT; triangles: single-energy 90 kVp PCD-CT. Symbols depict the mean of three measurements and the accompanying shaded boundaries depict the standard error of the mean.

CNR-measurements were very reproducible: relative SE < 7.0% with a median (range) of 2.1% [0.8–4.2], 1.3% [0.3–3.3] and 1.8% [0.5–7.0] for the EID, PCD at 120 kVp, and PCD at 90 kVp, respectively. Figure 2 demonstrates CNR in case no IR is applied as a function of the VMI energy level for all six combinations of phantom type and size. For energies below 60 keV, the CNR of the PCD-based images was generally higher (*p* < 0.05) than the CNR of the corresponding EID-based images. For energies below 70 keV, PCD-based images acquired at 90 kVp demonstrated a higher CNR than the images acquired at 120 kVp (Figure 2) (*p* < 0.05). For both the thorax and the abdomen phantom, CNR decreased with increasing phantom size. The highest CNR values, i.e., at 40 keV, for all phantom sizes for both the EID-based and the PCD-based scans are reported in Table 2.

**Figure 3:**
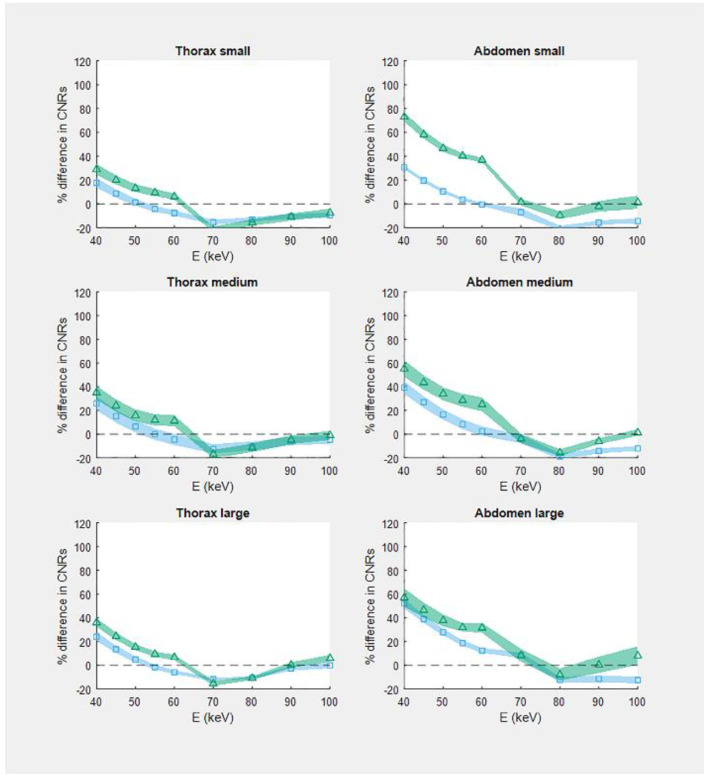
Relative CNR in virtual monoenergetic images reconstructed without iterative reconstruction technique as a function of energy E (keV). For each phantom and energy, CNR of the PCD-based system is presented relative to the CNR of the EID-based system. Squares: single-energy 120 kVp PCD-CT. Triangles: single-energy 90 kVp PCD-CT. Symbols depict the estimated value and the accompanying shaded boundaries depict the standard error of this estimate.

Figure 3 demonstrates the percentage difference in the CNR between EID- and PCD-based image reconstructions (without IR) as a function of monoenergetic energy for all six phantoms. Compared to the EID-based system, the CNR at 40 keV of the PCD-based system was up to 52% higher (in the large abdomen phantom) and up to 73% higher (in the small abdomen phantom) for scans at 120 kVp and 90 kVp, respectively. Although the CNR was higher in the thorax phantom, the percentage difference in CNR between EID and PCD was higher for the abdomen phantom. 

**Figure A1:**
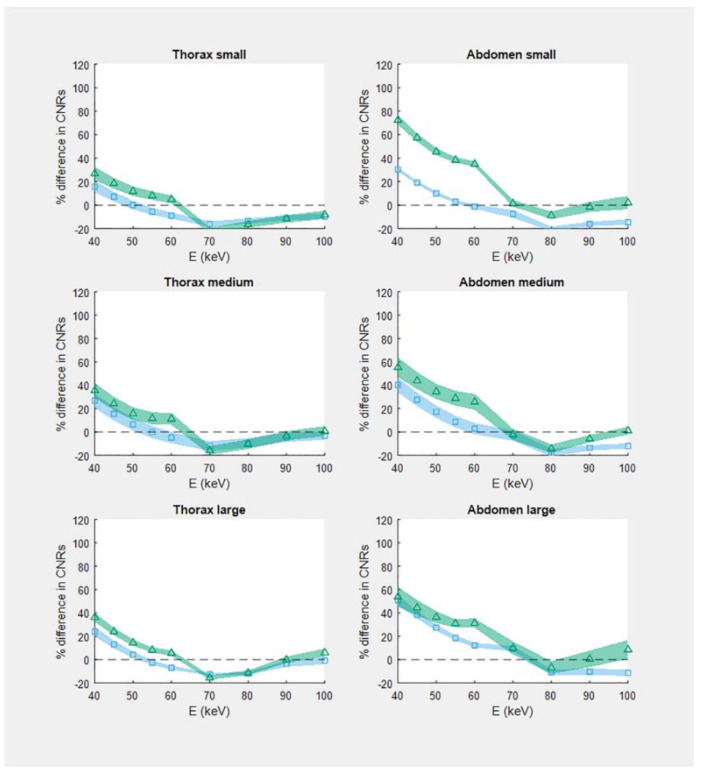
Relative CNR in virtual monoenergetic images reconstructed with iterative reconstruction (IR) technique as a function of energy E (keV). For each phantom and energy, CNR of the PCD-based system (with 50% IR strength) is presented relative to the CNR of the EID-based system (with 60% IR strength). Squares: single-energy 120 kVp PCD-CT. Triangles: single-energy 90 kVp PCD-CT. Symbols depict the estimated value and the accompanying shaded boundaries depict the standard error of this estimate.

**Figure A2:**
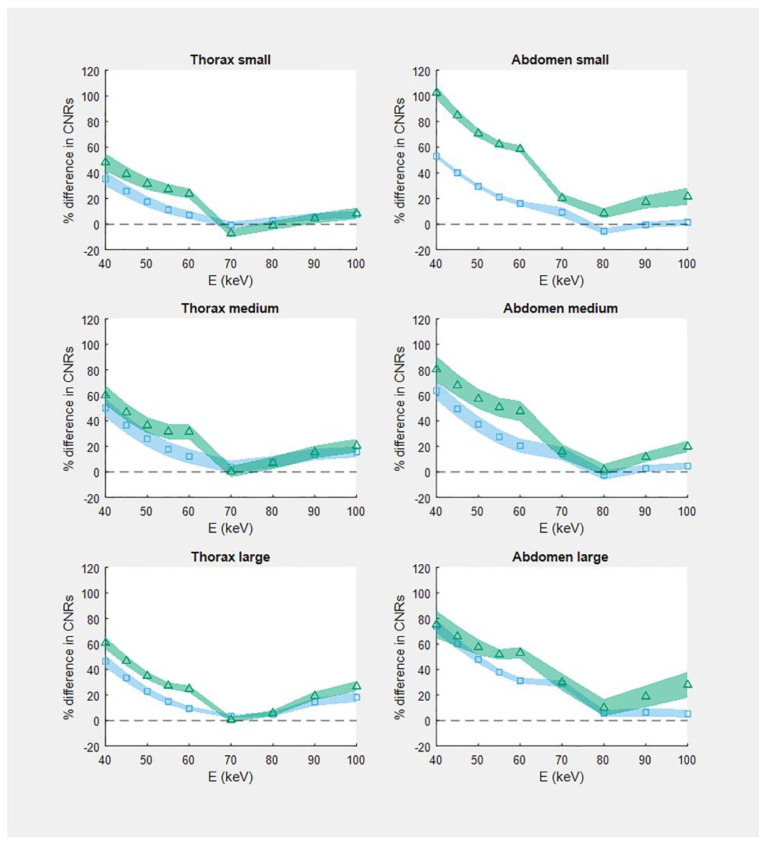
Relative CNR in virtual monoenergetic images reconstructed with iterative reconstruction (IR) technique as a function of energy E (keV). For each phantom and energy, CNR of the PCD-based system (with 75% IR strength) is presented relative to the CNR of the EID-based system (with 60% IR strength). Squares: single-energy 120 kVp PCD-CT. Triangles: single-energy 90 kVp PCD-CT. Symbols depict the estimated value and the accompanying shaded boundaries depict the standard error of this estimate.

Similar results were obtained when comparing the systems while applying IR techniques. EID-based images at 60% IR strength performed worse than PCD-based images at 50% IR strength, specifically at low monoenergetic levels and PCD-based scans at 90 kVp (Figure A1). The performance of the PCD-based system improved even further when using a 75% IR strength instead of 50% (Figure A2).

### 3.2. CT Values and Noise

**Figure 4:**
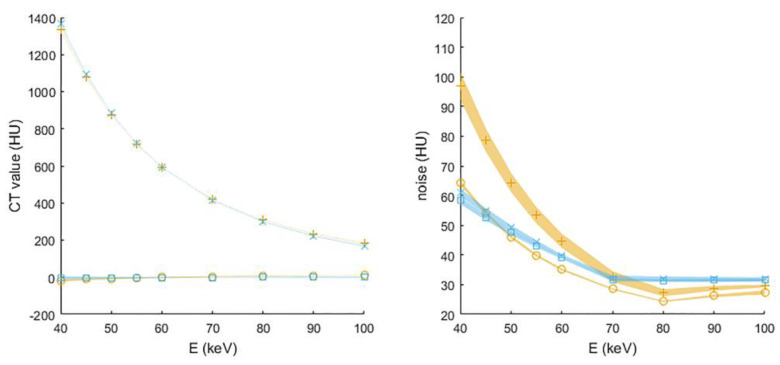
Mean (**left**) and standard deviation (**right**) of CT values (numbers expressed in Hounsfield units (HU)) in water and water with iodine as a function of virtual monoenergetic energy E (keV) for the medium sized abdomen phantom reconstructed without iterative reconstruction technique. For EID-based dual-energy data, circles and plus signs correspond to ROI’s in water and water with iodine, respectively. For single-energy 120 kVp PCD-CT, squares and crosses correspond to ROI’s in water and water with iodine, respectively. Symbols depict the estimated value based on three scans and the accompanying shaded boundaries depict the standard error of this estimate.

The mean CT values of water and water with iodine were similar for the EID-based and PCD-based VMI images reconstructed without IR (Figure 4). As expected, the CT value of water was constant for keV levels 40–100. In addition, as expected, the CT value of water containing iodine was highest for the lowest keV setting and decreased with increasing keV levels. 

Noise behavior differed between the EID- and PCD-based systems. In the EID-based system, noise levels were higher for the insert containing water and iodine than for the insert containing water only. In the PCD-based system, image noise was the same for both inserts with and without iodine. For both systems, image noise increased when lowering the monoenergetic energy level. 

## 4. Discussion

In this study, we evaluated the CNR of an iodinated contrast agent in VMI from both an EID-based and a PCD-based CT scanner. The optimal CNR was obtained with the PCD-based system operating at a tube voltage of 90 kVp in VMI reconstructions at 40 keV. To the best of our knowledge, this study is the first to evaluate iodinated contrast agent imaging with VMI on the first clinical photon-counting CT scanner by comparing it to dual-energy data acquired with a DSCT system. Recently, Rajendran et al. also discussed VMI with a photon-counting CT [16,22]. However, in these papers no comparison with VMI of EID-based dual-energy scans was made. Earlier, Gutjahr et al. described improved CNR in their research comparing PCD-CT to a conventional EID-CT [23]. Their comparisons were based on single-energy EID-CT only and thus did not include the CNR in VMI reconstructions. Leng et al. demonstrated that the CT number accuracy for VMI from their research on the PCD-CT scanner was comparable to that of EID-DSCT scanners, both reconstructed with the WFBP algorithm [24]. Their results are comparable to our results without IR. 

We quantified image noise by measuring the standard deviation of the CT numbers of both water and water with iodine, instead of water only. By doing so, we took into account the difference in noise magnitude between water and iodine. In our opinion, this is important when investigating lesion detectability. Consequently, a direct comparison with papers using other definitions of image noise is harder to make. Interestingly, for the PCD-based system, noise levels did not differ for water and iodine. This might be explained by the nature of the noise reduction algorithm applied. Most likely, this raw data-based technique is more advanced and improved compared to the image-based algorithms applied in EID-based systems [5] and thus may not be attributable to the use of PCD-technology alone.

Since the PCD system uses a new IR technique (QIR) and has four instead of five levels of IR strength, the VMI reconstructed with IR turned off was primarily used for the comparison between the PCD- and EID-based systems. Nevertheless, the image reconstruction techniques differ between the two systems, as discussed in the introduction. Moreover, IR is commonly used in clinical practice, and we have shown that the advancement in the IR techniques contributes to the superior performance of PCD-CT over EID-CT.

One of the practical advantages of acquiring VMI images with PCD instead of EID is within the acquisition part. Where the VMI of dual-source EID-based CT requires a relatively low pitch in combination with limited longitudinal coverage (38.4 mm instead of 57.6 mm), single- or dual-source (single tube voltage) on the PCD-CT operate at the full detector width (57.6 mm). Additionally, dual-source dual-energy EID-CT requires two different tube voltages, therefore sacrificing temporal resolution: 125 ms instead of 66 ms. In other words, VMI images are also available in PCD-CT examinations, where a high temporal resolution with dual-source acquisition is required, even at high pitch (3.2) with a scan speed of 737 mm/s. This enables the possibility to use VMI and spectral data, even for fast moving objects, such as in cardiac imaging by eliminating motion artifacts with the high temporal resolution of 66 ms. This makes it of great value in routine clinical imaging, even in challenging cases with non-cooperative patients such as intensive care unit patients or pediatric patients, where normally a breath-hold is used to freeze motion. Additionally, the VMI images are available with high-resolution imaging and can be combined with metal artifact reduction reconstruction algorithms, making it of interest for imaging all body regions. Thus, VMI obtained with a PCD-based system may facilitate the reduction in the contrast agent and radiation dose and improve lesion detection. It is important to realize that with the use of a tube voltage of 90 (instead of 120 kVp), the spectral analysis is limited to VMI only. Other spectral analyses such as virtual non-contrast and iodine mapping are not available. Therefore, the 90 kVp scan mode is especially ideal for imaging in iodine-enhanced CT exams where no additional spectral analysis is needed.

The results at 70 keV and above demonstrated that the CNR of the EID-based system was higher or similar to the PCD-based system. In general, image noise suppression in VMI images above the 70 keV is not required as much as it is for the lower keV levels, as attenuation of iodine contrast is higher and preferred at low tube voltage or low keV. 

Our study has some limitations that need to be considered. This study was phantom-based for a direct comparison between the two (high-end) systems. However, only one insert containing a relatively high iodine concentration was used that is representative for CT angiography scans in the arterial phase. Therefore, it was not possible to conclude whether the CNR of the PCD-system will also be superior at lower iodine concentrations found in the venous or late enhancement phase. A paper by Bette et al. investigated the enhancement of liver metastases and demonstrated the improvements in the use of VMI from PCD-CT in clinical use [25]. They concluded that CNR was preserved across a broad body mass index (BMI).

As stated above, the VMI reconstruction technique incorporates a noise reduction algorithm. This might affect the spatial resolution and detectability of lesions differently for the EID- and PCD-based systems. However, this was not investigated in this study. 

All protocols were configured to match the clinically-used dose levels. Results may vary per clinical application and depend on the radiation dose level applied. The next step might be to investigate whether the use of PCD-CT VMI reconstructions permit a radiation and/or contrast media dose reduction without compromising image quality and diagnostic confidence. 

## 5. Conclusions

Overall, single tube voltage PCD-based CT demonstrated increased CNR in virtual monoenergetic iodine imaging for low keV in comparison with VMI, based on an EID DSCT system operating at two different tube voltages. For the lower energy levels (<60 keV), PCD-CT at 90 kVp demonstrated the highest image quality with respect to CNR.

## Figures and Tables

**Table 1:** Acquisition and reconstruction parameters.

Scanner Name/Software Version	SOMATOM Force/Syngo CT VB10	NAEOTOM Alpha/Syngo VA40A_2.01
Detector type	energy integrating	photon counting
Scan mode	dual-source, helical	single-source, helical
Tube voltages (kVp)	80/Sn150 (thorax); 90/Sn150 (abdomen)	90 and 120 (both thorax and abdomen)
CTDIvol_32_ S/M/L thorax (mGy) *	2.2/3.7/6.9	2.2/3.7/6.9
CTDIvol_32_ S/M/L abdomen (mGy) *	3.4/5.4/8.1	3.4/5.4/8.1
Collimation (mm)	96 × 0.6 = 57.4	144 × 0.4 = 57.4
Pitch	0.8	0.8
Rotation time (s)	0.5	0.5
Image reconstruction kernel	Qr40	Qr40
Reconstruction technique **	ADMIRE strength 3 (60%); WFBP	QIR strength 2 (50%) and 3 (75%); QIR-off
VMI energy levels (keV) ***	40, 45, 50, 55, 60,70, 80, 90, 100	40, 45, 50, 55, 60,70, 80, 90, 100
Slice thickness/increment (mm)	1.0/1.0	1.0/1.0
Field-of-view (mm)	110	110
Reconstruction matrix size	512 × 512	512 × 512

* Typical CTDIvol values were determined for each phantom type and size (small/medium/large); no tube current modulation was used during scanning; ** Weighted-filtered backprojection (WFBP) corresponds with the iterative reconstruction technique turned off. Numbers between brackets correspond with the strength as percentage of the maximum strength available; *** VMI: Virtual monoenergetic images reconstructed with MonoEnergetic plus (Siemens Healthineers).

**Table 2:** Contrast-to-noise-ratios for virtual monoenergetic images at 40 keV of an energy integrating detector (EID) and a photon-counting detector (PCD)-based CT system. Iterative reconstruction technique was turned off.

	CT System		
Phantom Type and Size	EID	PCD at 120 kVp	PCD at 90 kVp
Thorax small	25.2 ± 0.7	29.6 ± 0.6	32.4 ± 0.7
Thorax medium	21.3 ± 0.9	26.8 ± 0.5	28.8 ± 0.1
Thorax large	20.0 ± 0.5	24.8 ± 0.5	27.2 ± 0.3
Abdomen small	18.3 ± 0.2	23.9 ± 0.1	31.7 ± 0.6
Abdomen medium	16.5 ± 0.6	22.9 ± 0.3	25.5 ± 0.7
Abdomen large	14.4 ± 0.2	21.9 ± 0.5	22.6 ± 1.1

Values given in mean ± standard error based on three repeated scans for each protocol.
